# Association between social integration and medical returns among the migrant elderly following children to Jinan City China

**DOI:** 10.1186/s12889-021-11901-7

**Published:** 2021-10-09

**Authors:** Jinfeng Zhao, Fanlei Kong, Shixue Li

**Affiliations:** 1 Centre for Health Management and Policy Research, School of Public Health, Cheeloo College of Medicine, Shandong University, Jinan, 250012 China; 2 NHC Key Lab of Health Economics and Policy Research (Shandong University), Jinan, 250012 China

**Keywords:** Migrant elderly following children, Medical return, Social integration, Medical insurance

## Abstract

**Background:**

Studies had shown that social integration was related to the utilization of medical services. Few studies investigated the relationship between social integration and medical returns among the elderly. None research had ever clarified the effect of social integration on medical returns among the migrant elderly following children (MEFC) to new cities. This study aimed to explore the association between social integration and medical returns among the MEFC in Jinan, China.

**Method:**

This cross-sectional study included 627 MEFC in Jinan China. Social integration was evaluated by economic integration, acculturation, and identification. Medical return was assessed by asking the subjects whether go back to hometown to use the medical services when ill. Chi-squared test and multivariable logistic regression were applied to analyze the association between social integration and medical returns of the MEFC.

**Results and discussion:**

It was found that 20.3% of the MEFC had a medical return. As for social integration, those who had not joined local medical insurance (OR = 3.561, 95% CI 1.577–8.039, *p* = 0.002) and were unwilling to stay for a long time (OR = 2.600, 95% CI 1.620–4.174, *p* = 0.001) were more likely to have a medical return. Furthermore, our findings showed that the MEFC who were accompanied by one or more (OR = 1.568, 95% CI 1.027–2.392, *p* = 0.037) were more likely to have a medical return than those who migrated alone.

**Conclusion:**

Negative relationship between social integration and medical returns was found among the MEFC, which means the better social integration of the MEFC would generally have fewer medical return, as well as the better refunding connections of the medical insurance between the current residence and hometown.

## Background

China’s population migration has entered a stage characterized as family migration.

Moreover, due to population aging and the traditional culture highlighting family union and intergenerational mutual support, an increasing number of elderly people are migrating with their children [1]. Statistically, the number of internal migrant elderly has increased rapidly at an average annual rate of 6.6% since 2000, reaching 13.4 million in 2015 [2] . The rapid growth of the number and life expectancy of the migrant elderly has made relevant research more and more important.

In this study, the migrant elderly following children (MEFC) to new cities refers to those who are 60 years old or over and who migrated from their original residence with their children to new cities [3]. The main reasons for their migration include caring for the younger generation and providing care for the elderly. Compared with younger migrants (working-age population under 60 years), the MEFC have gone through various stages of life (birth, employment, and education), entering into the later stages of life [4]; their physiological function and resistance are reduced, coupled with increasing morbidity and medical treatment rates [5]. Synonymous with the foreign migrant elderly [6, 7], the migrant elderly in China are also faced with environmental discomfort, inadequate pension services, and other related issues. Therefore, the MEFC, with dual vulnerable attributes of a migrant population and an elderly population [8] has a higher demand for medical services. This made how the MEFC’s health status is and how about MEFC’s medical services usage become an important public health problem.

Research showed that [9] improved integration helps migrants create social capital and promote their mental health. Policies have been formulated to promote the health of the migrant population in many international organization and countries. In 2015, the United Nations listed social integration as one of its sustainable development goals ensuring that vulnerable groups (including migrants) have fair access to basic services and the promotion of healthy lives for the entire population [10]. The World Health Organization called for practical measures to establish a health system that is friendly to immigrants and refugees in protecting their health [11]. As early as 2009, the Chinese government launched a national basic public health service program for all Chinese residents (migrants included) free of charge and the number of free public health services has been increased to 14 projects so far [12]. Nevertheless, previous studies have demonstrated that the consultation rate and main medical service resource utilization rate of the migrant population in the inflow areas were still fairly low [13]. Meanwhile, many of the migrant population prefer returning to their hometown for medical services [14, 15].

The United Nations [16] sustainable goal for social integration encourages an environment where people are fully respected for their dignity, have common interests, and have equal opportunities to participate in society, culture, economy, and politics. The European Union defined [16, 17] social integration as the process of ensuring that groups at risk and social exclusion obtain the necessary opportunities and resources, and through full participation in economic, social, and cultural life, can enjoy a normal life and the normal social welfare in the society where they live. Chinese scholar Qiao Nan defined [18] social integration as the process of gradual assimilation and reduction of exclusion between the migrant population and the local population. In addition, new migrants should enjoy the necessary rights in the inflow area and actively participate in the development of the inflow area. Social integration in this study refers to the process by which the migrant population integrates into the influx place in terms of residence, employment, values and lifestyle, gradually enjoying and fulfilling the same rights, benefits, and obligations as local residents. Although the definition and measurements of social integration are not completely cohesive, previous studies showed certain similar dimensions: economic integration, acculturation, and identification [19–21]. The report sponsored by the National Health Commission of China showed that the overall social integration level of the migrant population was fairly low in China, although positive increase was also observed [22].

Some studies explored the determinations of social integration among migrant populations [23, 24], as well as the association between social integration and physical or mental health [25, 26]. Previous research showed that social integration of migrants had a significant association with health and medical services utilization [27–29]. The Swedish government regarded improving the social integration of migrants as an important mental health promotion strategy to minimize the mental health gap caused by migration [30]. A study by De Jesus et al. regarding cross-border health care utilization of immigrants in the U.S. found that the lack of local medical insurance and language barriers were important factors for the Hispanic American population in returning to Mexico for medical services [31].

At present, there are many studies [32–34] on the factors affecting the medical care-seeking behaviors of the migrant population. However, there are limited relevant studies about the migrant elderly population. Most of these references [13, 35, 36] showed that illness or health status is an important factor affecting medical care-seeking behaviors of the migrant elderly population.

To date, very few studies examined the relationship between social integration and medical returns (returning hometown for medical services) among the young and middle-aged migrants; no study explored the above relationship among the MEFC. This study aims to investigate the relationship between social integration and medical returns among the MEFC in Jinan, China in order to fill this research gap.

## Methods

### Data collection and participants

The data were collected in Jinan City, Shandong Province, China, in August, 2020. By the end of 2019, the local resident population was 8.91 million, an increase of 0.78% over the previous year, while the registered population was 7.98 million, an increase of 1.46% [37]. There were 2.9 million migrants in Jinan City in 2019 [38]. The migrants above aged 60 years and followed their children to Jinan City were the participants of this study. We used multi-stage cluster sampling to select the participants for the study.

In the first stage, three out of ten districts were chosen as the primary sampling units (PSUs), considering geographic location and economic development. In the second stage, three sub-districts were chosen from each PSU as the secondary sampling units (SSUs), which meant that one sub-district was chosen from each of the previously selected district. In the third stage, three communities were selected from the SSUs, which meant that one community was chosen from each of the previously selected sub-districts.

Thirty-two university students became investigators after training on purpose of the study, the contents of the questionnaire, and the social survey technique. Eleven of the investigators were from Shandong University, thirteen from Jinan University, two from Dongying Vocational Institute, and seven from Weifang Medical University. The investigators conducted twenty-minute, face-to-face interviews, to collect the data. Initially, 670 elderly migrants who followed their children were chose and interviewed. However, 14 of them were excluded from the sample because of obvious logical errors in the questionnaire or incomplete questionnaires. Finally, we collected 656 valid questionnaires. Moreover, 29 of them never visited a hospital/ pharmacy shop within a year. As a result, 627 MEFC were eventually included in the analysis.

### Variables

#### Dependent variable

Medical return was accessed by asking the participants, “Where do you get treatment for sickness/disease?” There were two options for the question above, “Go back to the hospital in the hometown” and “Go to the hospital in the current residence.”

#### Independent variables

##### Social integration

Based on existing literature [19–21], this study used three dimensions to measure social integration: economic integration, acculturation, and identification. Firstly, the indicator of local medical insurance status (joined and not joined) was used to represent the dimension of economic integration. Considering that, most of the MEFC had withdrawn from the labor market and lived with their children, common employment, housing conditions and income were not included in our economic integration measures. Secondly, acculturation integration referred to the level of understanding, recognition, and acceptance of the local culture (such as language, diet, and other regional cultures) by the MEFC. It was usually measured by indicators such as local language use and behavioral practices [39]. A previous study [40] showed that the length of mobility has a positive impact on the acculturation integration. Therefore, combined with the questionnaire in the study, this study used “duration of stay in current residence (five years and below, and more than five years)” to evaluate the dimension of acculturation. Finally, identification reflected the sense of belonging of the MEFC to the place where they migrated. Using “willingness to stay for a long time (no and yes)” to express the dimension of identification in this study which was also adopted in Zhang’s study on migrant seniors in China [41].

##### Social demographic characteristics

Based on previous studies [42, 43] and our questionnaire, the social demographic characteristics included age, gender, ethnic group, marital status, education level, hukou (a household registration system used in China), family member of current residence, migration reason, migration range, temporary resident permit, and the number of accompanying migrants. The social demographic characteristics were described and classified as follows: age was divided into two groups (60–65,≥66), ethnic group (Han, and ethnic minority group), marital status including married and single (divorced, widowed), an education level (uneducated, primary school, middle school, others), family members of current residence (less than five, five, more than 5 years), migration reason (taking care of grandchildren, curing a disease or rehabilitation, others), migration range (trans-county, trans-city, trans-province, or county), temporary residence permit (yes, no), and the number of accompanying migrants including alone and accompanied by one or more.

##### Disease

The disease included common diseases of the elderly, including hypertension disease, diabetes, heart disease, cerebrovascular diseases, cancer, lumbago, knee pain, and headache.

### Statistical analysis

Data were analyzed using SPSS 24.0, and statistical significance was set as *p*< 0.05. Percentages of nominal variables were determined. First, a Chi-square test was performed to explore the relationship between the related variables and medical returns. Three multivariable logistic regression models with an enter method examined the associations between social integration and medical return among the MEFC. In Model 1, only sociodemographic characteristic factors were included. In Model 2, only common diseases of the elderly were included. In Model 3, we included sociodemographic factors and diseases factors with a statistically significant impact on the dependent variables in Model 1 and Model 2 as control variables. This step tested whether social integration had a significant impact on MEFC’s medical care-seeking behaviors when controlling for other potential confounding factors.

## Results

### Participants’ demographic characteristics

Table 1 shows the sociodemographic information of the 627 MEFC. About 20.3% the MEFC chose to have a medical return. More than half of them were female (400, 63.8%), in the age group of 66 years and above (320, 51%), married (560, 89.3%), Han (620, 98.9%), lower than primary school (323, 51.5%), living with five family members in current residence (240, 38.3%), and had rural hukou (548, 87.4%). The proportions from low to high for four income groups were 19.8, 23.6, 27.4, and 29.2%, respectively. Furthermore, most of the MEFC moved to take care of their grandchildren (418, 66.7%), have not applied for temporary resident (405, 64.6%) and were accompanied by one or more (410, 65.4%). According to the results of Chi-square test analysis, factors including “whether to apply for a temporary permit” and “the number of accompanying migrants” were significantly associated with medical returns among the MEFC.

**Table 1:** The sociodemographic characteristic of the MEFC

Variables	N(%)	Medical return		χ^**2**^	***p***
		No	Yes		
**Observations**	627(100)	500(79.7)	127(20.3)		
**Age**	66.2 ± 4.6^a^	500(79.7)	127(20.3)	24.285	0.560
**Gender**				0.167	0.682
Male	227(36.2)	183(80.6)	44(19.4)		
Female	400(63.8)	317(79.3)	83(20.8)		
**Ethnic group**				0.156	0.693
Han	620(98.9)	494(79.7)	126(20.3)		
Minority ethnic^b^	7(1.1)	1(14.3)	6(85.7)		
**Marital status**				0.684	0.408
Married	560(89.3)	444(79.3)	116(20.7)		
Single	67(10.7)	56(83.6)	11(16.4)		
**Education level**				0.971	0.808
Uneducated	187(29.8)	151(80.7)	36(19.3)		
Primary school	136 (21.7)	108(79.4)	28 (20.6)		
Middle school	183(29.2)	142(77.6)	41(22.4)		
Others	121(19.3)	99(81.8)	22(18.2)		
**Household monthly income**				3.603	0.308
Q1^c^	148(23.6)	116(78.4)	32(21.6)		
Q2	183(29.2)	145(79.2)	38(20.8)		
Q3	124(19.8)	94(75.8)	30(24.2)		
Q4	172(27.4)	145(84.3)	27(15.7)		
**Hukou**				0.808	0.369
Rural	548(87.4)	43(79.2)	114(20.8)		
Urban	79(12.6)	66(83.5)	13(16.5)		
**Family members in current residence**				0.431	0.806
≤ 4	202(32.2)	158(78.2)	44(21.8)		
5	240(38.3)	193(80.4)	47(19.6)		
≥ 6	185(29.5)	149(80.5)	36(19.5)		
**Migration reason**				4.739	0.094
Taking care of grandchildren	548(87.4)	433(79.0)	115(21.0)		
Curing a disease or rehabilitation	26(4.1)	19(73.1)	7(26.9)		
Others	53(8.5)	48(90.6)	5(9.4)		
**Migration range**				2.050	0.359
Trans-county^d^	143(22.8)	112(78.3)	31(21.7)		
Trans-city^e^	418(66.7)	331(79.2)	87(20.8)		
Trans-province or county^f^	66(10.5)	57(86.4)	9(13.6)		
**Applied for temporary resident permit**				11.006	0.001^***^
Yes	222(35.4)	193(86.9)	29(13.1)		
No	405(64.6)	307(75.8)	98(24.2)		
**The number of accompany-migrated**				12.678	0.001^***^
Alone	217(34.6)	156(71.9)	61(28.1)		
Accompanied by one or more	410(65.4)	344(83.9)	66(16.1)		

Notes: *p*<0.05,^**^p<0.01,^***^p<0.001

^a^Presented as mean ± standard deviation

^b^Minority ethnic including Manchu, Hui, Mongol, Tibetan and other minority ethnic groups

^c^Quartile 1 (Q1) is the lowest and Quartile 4 (Q4) is the highest group

^d^Trans-county refers to Chinese internal migrant flowing into the urban area from a county or village in Jinan

^e^Trans-city refers to Chinese internal migrant flowing into Jinan from another city in Shandong Province

^f^Trans-province refers to Chinese internal migrant flowing into Jinan City of Shandong Province from other provinces

### Participants’ diseases

Table 2 shows the diseases of the 627 MEFC. The common chronic diseases, like tumors and pain are less among the MEFC; 25.4% are hypertension patients, 25.5% diabetic patients, and 20.8% heart disease patients who had a medical return.

**Table 2:** The diseases and medical return of the MEFC

Variables	N(%)	Medical return		χ^**2**^	***p***
		No	Yes		
**Observations**	627(100)	500(79.7)	127(20.3)		
**Hypertension**				2.581	0.108
Yes	126(20.1)	94(18.8)	32(25.4)		
No	501(79.9)	406(81.2)	95(74.8)		
**Diabetes**				0.942	0.332
Yes	51(8.1)	38(74.5)	13(25.5)		
No	576(91.9)	462(80.2)	114(19.8)		
**Heart disease**				0.005	0.943
Yes	24(3.8)	19(79.2)	5(20.8)		
No	603(96.2)	481(79.8)	122(20.2)		
**Cerebrovascular disease**					0.018^**^
Yes	52(8.3)	48(92.3)	4(7.7)		
No	575(91.7)	452(78.6)	123(21.4)		
**Cancer**					0.106
Yes	40(6.4)	36(90.0)	4(10.0)		
No	587(93.6)	464(73.1)	123(21.0)		
Others	53(8.5)	48(90.6)	5(9.4)		
**Lumbago**				8.704	0.003^**^
Yes	78(12.4)	72(92.3)	6(7.7)		
No	549(87.6)	428(78.0)	121(22.0)		
**Knee pain**				0.858	0.354
Yes	41(6.5)	35(85.4)	6(14.6)		
No	586(93.5)	465(79.4)	121(20.6)		
**Headache**					0.032^**^
Yes	31(4.9)	29(93.5)	2(6.5)		
No	596(95.1)	471(79.0)	125(21)		

*p*<0.05,^**^p<0.01,^***^p<0.001

### Social integration status

Social integration was indicated through economic integration (local medical insurance status), acculturation (duration of stay in current residence), and identification (willingness to stay for a long time). Regarding economic integration, 514 (82%) migrants joined the local medical insurance (Table 3). Regarding acculturation, more than half the MEFC were in their current residence for less than 5 years (54.1%); as for identification, 52% of the participants were willing to stay for a long time in their current residence. Chi-square test analysis results showed that factors including “local medical insurance status,” “duration of stay in their current residence,” and “willingness to stay for a long time” were statistically significantly associated with medical returns among the MEFC.

**Table 3:** Medical returns across different dimensions of social integration among MEFC

Variables	N(%)	Medicine return		χ^**2**^	p^*^
		NO	Yes		
**Observations**	627(100)	500(79.7)	127(20.3)		
**Economic integration status**					
*Joining local medical insurance*				16.871	0.001^***^
Yes	113(18)	106(93.8)	7(6.2)		
No	514(82)	394(76.7)	120(23.3)		
**Acculturation**					
*Duration of stay in current residence*				9.350	0.002^**^
≤ 5 years	339(54.1)	255(75.2)	84(24.8)		
>5 years	288(45.9)	245(85.1)	43(14.9)		
**Identification**					
*Willingness to stay for a long time*				32.567	0.001^**^
Yes	326(52.0)	284(87.1)	42(12.9)		
No	201(32.1)	134(66.7)	67(33.3)		
Not sure	100(15.9)	82(82.0)	18(18.0)		

*p*<0.05,^**^p<0.01,^***^p<0.001

### Association between social integration status and medical returns

Table 4 shows the results of the three models by logistic regression analysis. Only social integration status was included in Model 1, and the results of the multivariable logistic regression analysis indicated that the relationship between the two aspects of social integration and medical returns among the MEFC was statistically significant. Specifically, the MEFC who did not join local medical insurance (*p* = 0.001, OR = 3.904), had no willingness to stay in their current residence for a long time (*p* = 0.001, OR = 2.795) preferred medical returns. In Model 2 and Model 3, after including social demographic characteristics factors and disease factors, the results still showed that there was a statistically significant association between the two aspects of social integration and medical returns.

**Table 4:** Association between social integration and medical returns among the MEFC

Variables	Regression Model 1			Regression Model 2			Regression Model 3		
	OR	95%CI	*p*	OR	95%CI	*p*	OR	95%CI	*p*
**Joining local medical insurance**									
Yes	1.0			1.0			1.0		
No	3.904	1.750–8.709	0.001^**^	3.450	1.535–7.754	0.003^**^	3.561	1.577–8.039	0.002^**^
**Duration of stay in current residence**									
≤ 5 years	1.0			1.0			1.0		
>5 years	0.662	0.432–1.015	0.059	0.714	0.462–1.102	0.128	0.731	0.470–1.134	0.162
**Willingness to stay for a long time**									
Yes	1.0			1.0			1.0		
No	2.795	1.778–4.392	0.001^**^	2.491	1.563–3.968	0.001^**^	2.600	1.620–4.174	0.001^***^
Not sure	1.288	0.697–2.381	0.419	1.187	0.637–2.212	0.586	1.258	0.670–2.360	0.475
**Applied for temporary resident permit**									
Yes				1.0			1.0		
No				1.385	0.853–2.249	0.188	1.366	0.837–2.229	0.212
**The number of accompany-migrated**									
Alone				1.0			1.0		
Accompanied by one or more				1.555	1.025–2.360	0.038^**^	1.568	1.027–2.392	0.037^**^
**Cerebrovascular disease**									
Yes							1.0		
No							1.975	0.649–6.006	0.230
**Lumbago**									
Yes							1.0		
No							3.208	1.302–7.903	0.011^**^
**Headache**									
Yes							1.0		
No							1.720	0.358–8.270	0.498

*p*<0.05,^**^p<0.01,^***^p<0.001

Furthermore, the number of accompanying migrants had a significant association with medical returns. The MEFC who were accompanied by one or more were more likely to have a medical return than those who migrated alone (*p* = 0.037, OR = 1.568). The MEFC with no waist pain were more willing to have a medical return than those with waist pain (*p* = 0.011, OR = 3.208) (Table 4).

## Discussion

In this study, 20.3% of the MEFC had a medical return. A study based on national migrant population data of China in 2014 pointed out that 22.5% of the migrant elderly have a medical return, which was slightly higher than the current study [44]. The reason may be due to the increasing attention paid by various departments of the Chinese government to the problem of migrants’ seeking medical care in recent years [36]. Our result is also lower than the rate of medical returns (63.2%) for migrant workers in Beijing, 2008 [43]. There are some possible reasons for this. Although Beijing, the capital city of China, is superior to Jinan in terms of economy and medical conditions, problems will arise at the same time. Firstly, compared with Jinan City, the waiting cost for obtaining medical services in Beijing is higher, meaning longer time for registered appointments, higher price on accommodation and higher medical expenses [42]. Secondly, compared with the MEFC who live with their families in the inflow cities, migrant workers who returned to their hometowns for medical services when sick could get care and comfort from their families. Thirdly, compared with most MEFC who need to take care of their grandchildren, migrant workers are more substitutable for factories, which made their medical return easier. When considering taking care of their grandchildren, many MEFC may choose to tolerate the disease and quit the medical return.

Regarding social integration, a negative relationship between social integration and medical returns among the MEFC was found. The better social integration among the MEFC, the fewer medical returns. Specifically, this negative relationship was manifested in the two dimensions of social integration: economic integration and identification.

Firstly, the MEFC who have not joined the local medical insurance are more likely to return to their hometown for medical services. It is consistent with previous studies on internal migrants [42, 45, 46] and international immigrants [47–49]. At present, there is still a gap in China’s basic medical insurance program. When the medical service location is not the same as the insured area, the reimbursement proportion of medical expenses is low, and there are various cumbersome reimbursement processes [45]. Therefore, to get more compensation for medical expenses, coupled with the consideration of being more familiar with their hometown’s medical and health system, the elderly without local medical insurance would be more willing to have a medical return.

Some studies showed that the household monthly income was associated with the medical treatment places for the migrant population [42, 46, 50], which is inconsistent with our results. One possible reason for this was that the household monthly income we surveyed only includes the monthly income of the elderly and their spouses; their children’s income was not included in the household monthly income, and most of the MEFC did not know their children’ s income during the questionnaire survey. About 87.4% (548/627) of the MEFC were from rural areas and 80.7% (506/627) of them were lower than high school, indicating most of them were economically disadvantaged. As a country strongly influenced by the culture of filial piety [51], children will easily support the living expenses of the elderly. This may imply that most of the MEFC are economically disadvantaged and rely more on their children for medical care.

Secondly, the results showed that the MEFC who stated their willingness to stay in the inflow area for a long time have lower possibility on medical returns. Their willingness to stay long in the inflow area implies that migrants like the inflow areas, are willing to make friends with local residents, and have a stronger sense of belonging to the inflow cities [31, 52]. The MEFC can rely on help when looking for access to healthcare or when needing help in times of bad health conditions. Therefore, only when the MEFC have a broader social support and a stronger sense of intimacy in the inflow area, can they be less willing to have a medical return.

It is worth noting that the association between duration of stay in current residence and medical returns in univariate analysis was statistically significant, but after other factors were included, the association was no longer significant. The reason may be that economic integration has a greater impact on choosing medical places than a cultural adaptation for the MEFC. The residence duration of the MEFC was closer related to the time that they spent caring for their grandchildren in this study. Therefore, they are passive adapters to the local culture. Regardless of how long the MEFC stayed in their new residence, the amount for medical expenses will remain an important consideration in choosing medical places. This may be why the significant effect of cultural adaptation on the MEFC medical returns is different from other studies.

In addition to social integration, the number of accompanying migrants had a significant impact on medical returns. The MEFC who were accompanied by one or more were more likely to have a medical return than those who migrated alone. The reasons may be as follows: considering that children cannot take care of their young grandchildren due to work, the MEFC who migrated alone can only choose to seek medical care nearby so as to continue to look after their grandchildren. When other people (such as their spouse) migrated together, the sick MEFC were more likely to return their hometown because of the basic medical insurance.

The impact of temporary resident permits on medical returns was no longer statistically significant in the multivariate regression analysis. The association between the application of residence permits and the willingness to stay for a long time was found in a previous study [53]. Nevertheless, we could not eliminate the concurrent effect of economic factors. The elderly who apply for residence permits in their current residence enjoy the same basic public health services and free bus services as local residents. However, these benefits were relatively small and could not offset the problems caused by the reimbursement of medical insurance in their hometown.

The MEFC with waist pain were more willing to stay Jinan for medical services. Waist pain is very common in the elderly [54]. After consulting the reference [54], we learned that the daily activities of the elderly were not restricted when it was mild and were often ignored. In this situation, as children are affected by traditional filial piety, they were more willing to keep their parents around to facilitate care and ease their psychological worry [55].

It is noted that the effect of the better social integration on the fewer medical returns cannot be achieved without the effect of social support. The MEFC’ social contacts in the inflow cities are mainly government, community, families and neighbors [56]. An open and inclusive urban society is more helpful to promote the social integration of the MEFC. The government can implement some integration policies that are friendly to the migrant population, such as improving healthcare policy. The communities and neighbors in the inflow cities can provide a variety of useful information and timely help for the MEFC. In addition to necessary financial support, family members could enhance their confidence in living in other places by giving them more companion and understanding in daily life. All these supports above play an important role in improving social integration. And finally, these supports will help to improve the health status, well-being and life satisfaction of the MEFC [57].

### Limitation

Firstly, there is no unified indicator to assess social integration. Therefore, according to references and questionnaires, three dimensions including four questions were used to assess social integration in this study, which may have a bias of reliability and validity. Secondly, the specific indicators of social integration in this study may stay at the level of individual integration and it lacks of the group-level measurement indicators which should be improved in the future study. Thirdly, the little existed research on MEFC made the authors difficult to assessment the level of the social integration. To design a reasonable scale on measuring the social integration of MEFC becomes the future research issue in this study field. Fourthly, due to the COVID-19 pandemic, we only completed the questionnaire survey in Jinan and failed to start the survey in Shanghai as planned. This additional location may provide more information on MEFC in China, and provide a clearer picture. Finally, this study was a crossed-sectional design, so causality cannot be determined.

## Conclusion

It was found that nearly 23% of the MEFC returned to their hometown for medical services. In terms of integration of economy and identity, the better social integration of the MEFC, the fewer medical returns. The MEFC in Jinan who joined local medical insurances were willing stay for a long time and were less likely to return their hometown for medical services. Additionally, the number of accompanying migrants was also associated with the medical return. Measures that are more effective should be taken to promote the connections of the medical services utilization between the current residency and hometown, as well as the social integration of MEFC.

## Data Availability

Under reasonable requirements, the data and material of this study can be obtained from the corresponding author. The data are not publicly available due to privacy restrictions.

## Abbreviations

MEFC: Migrant elderly following children
OR: Odds ratio
CI: Confidence interval
